# Use of Computed Tomography of the Head in Patients With Acute Atraumatic Altered Mental Status: A Systematic Review and Meta-analysis

**DOI:** 10.1001/jamanetworkopen.2022.42805

**Published:** 2022-11-18

**Authors:** Roshan Acharya, Smita Kafle, Dhan Bahadur Shrestha, Yub Raj Sedhai, Meera Ghimire, Kishor Khanal, Queen Baba Malla, Uttam Nepal, Rojina Shrestha, Badri Giri

**Affiliations:** 1 Division of Pulmonary and Critical Care Medicine, Virginia Tech Carilion School of Medicine, Carilion Roanoke Memorial Hospital, Roanoke; 2 Department of Nursing, Fayetteville State University School of Nursing, Fayetteville, North Carolina; 3 Department of Internal Medicine, Mount Sinai Hospital, Chicago, Illinois; 4 Division of Pulmonary and Critical Care Medicine, University of Kentucky, Bowling Green; 5 Department of Internal Medicine, Cape Fear Valley Medical Center, Fayetteville, North Carolina; 6 Department of Critical Care Medicine, Nepal Mediciti Hospital, Kathmandu, Nepal; 7 Department of Internal Medicine, Nepalgunj Medical College, Kohalpur, Nepal; 8 Department of Internal Medicine, Kist Medical College, Lalitpur, Nepal

## Abstract

**Importance:**

The usefulness of computed tomography of the head (CTH) in patients with acute-onset atraumatic altered mental status (AMS) is poorly understood, but use in these patients remains high.

**Objective:**

To evaluate the use of CTH (event rate) in patients with AMS and the positive outcome event rate of the performed CTH studies.

**Data Sources:**

The PubMed/MEDLINE, PubMed Central, Embase, and CINAHL databases were searched using predefined Boolean parameters. All studies that met inclusion criteria until January 31, 2022, were included.

**Study Selection:**

Randomized clinical trials and observational, cohort, and case-control studies were included. Conference abstracts, reviews, letters, case reports, case series, systematic literature, and meta-analyses were excluded.

**Data Extraction and Synthesis:**

The systematic literature review was performed per Preferred Reporting Items for Systematic Reviews and Meta-analyses guidelines. Data were independently extracted by 2 authors. Data were pooled using a random-effects method.

**Main Outcomes and Measures:**

Event rate of CTH use in patients with acute atraumatic AMS. The CTH event rates and positive CTH event rates were calculated with 95% CIs.

**Results:**

Of 9338 studies identified, 26 qualified for the systematic review and 25 for the meta-analysis. The 25 studies in the meta-analysis included a total of 79 201 patients. The CTH event rate was 94% (proportion, 0.94; 95% CI, 0.76-1.00), and the positive CTH event rate was 11% (proportion, 0.11; 95% CI, 0.07-0.15). There was significant heterogeneity among the studies included (*I*^2^ > 50%, *P* < .001), for which a random-effects model was used. There was significant publication bias, as evident by an asymmetric funnel plot. There was no fluctuation of the results during the sensitivity analysis, which reassured the reliability of the data.

**Conclusions and Relevance:**

In this meta-analysis, CTH use among patients with acute-onset atraumatic AMS was very high with a low yield. Large-scale studies are needed to guide clinical decision-making in such a situation.

## Key Points

**Question:**

What is the proportion of use of noncontrast computed tomography of the head (CTH) in patients with acute-onset atraumatic altered mental status (AMS)?

**Findings:**

This systematic review and meta-analysis of 25 studies with 79 201 patients found that the use of CTH in patients with AMS was very high, but the positive yield on those CTH studies was low.

**Meaning:**

In this study, the use of CTH in patients with AMS was exceedingly high without a substantial yield.

## Teaser

This systematic review and meta-analysis assesses the use of noncontrast computed tomography of the head in patients with atraumatic acute-onset altered mental status.

## Introduction

Since the development of computed tomography (CT) in the 1970s, the use of CT studies has been increasing exponentially. In the mid-2000s, it was estimated that 60 million CT studies were performed annually in the US, which increased to an estimated 80 million CT studies in the late 2000s.^1,2^ A single health system study^1^ performed to see the trends of CT study use during a decade revealed that the proportion of CTs ordered in the emergency department (ED) increased by 81% (from 41.4% in 2000 to 74.4% in 2010). In contrast, the total ED patient volume remained stable. The reasons for the increasing trend are multifactorial, but the easy availability of the study and the prompt result are among the top reasons.^2,3^

In clinical practice, when a patient with acute altered mental status (AMS) is encountered, a CT of the head (CTH) study is usually performed as a part of the workup. The American College of Radiology 2019 appropriateness criteria for AMS do not explicitly favor CTH in patients with AMS without trauma, the risk for intracranial bleeding (such as anticoagulation use), hypertensive emergency, known intracranial process (mass, recent hemorrhage, recent infarct, central nervous system infection), new-onset delirium, or psychosis. The decision to perform CTH is left to the discretion of the physician.^4^ Because of ease and speed of execution, CTH is the preferred modality of radiologic investigation. In a previous study, CTH was ordered for almost half of the patients who presented with AMS and confusion in the ED, with a yield of only approximately 9%.^5^ Similarly, in another study, an estimated one-third of the CT studies were performed on the head, and 75% were performed in the hospital setting.^2^ Some studies suggest that the use of CTH has increased markedly in the US during the past 2 decades.^1,2^ However, CTH has a low yield in the evaluation of patients with AMS and adds to the cost of care along with radiation exposure.^5^ It is estimated that individuals with 3 to 4 lifetime CT studies have similar cancer risks to that of nuclear bombing survivors in Japan,^2^ but health care professionals remain unaware of this risk.^6^ Thus, we performed this systematic review and meta-analysis to fully appraise the available data. We assessed the proportion of noncontrast CTH use in patients with atraumatic acute-onset AMS. Furthermore, we explored the findings of positive CTH studies and their association with change in clinical management.

## Methods

### Search Strategy

The systematic review was conducted according to the Preferred Reporting Items for Systematic Reviews and Meta-analyses (PRISMA) reporting guideline.^7^ We searched the PubMed/MEDLINE, PubMed Central, Embase, and CINAHL databases. The Boolean parameters that were used to search AMS were [*altered mental status* OR *confusion* OR *disorientation* OR *unconscious* OR *AMS*]. Similarly, for CTH, the parameters were [*Tomography, X-ray Computed* OR *CT Head* OR *CTH*] (eTable 1 in the Supplement). We included studies published before January 31, 2022. The study's protocol was registered in PROSPERO (CRD42022324211).

### Eligibility Criteria and Outcomes

Randomized clinical trials and observational, cohort, and case-control studies were included. Conference abstracts, reviews, letters, case reports, case series, systematic literature, and meta-analyses were excluded. Patients having acute AMS, confusion, loss of consciousness, or disorientation without evidence of head trauma or focal neurologic deficits in the ED or while being admitted to the inpatient unit or intensive care unit (ICU) were eligible for the study. Studies with mixed age groups were included but were excluded if the study was performed with pediatric age groups only (<18 years of age). Outcomes included acute ischemic stroke, acute intracranial hemorrhage, intracranial mass, cerebral edema, and new identifiable lesions in the CTH result.

### Data Extraction

Studies were identified and screened for eligibility by 2 authors (R.A. and S.K.) independently based on inclusion criteria. EndNote software was used to maintain the records of identified and screened studies and to remove duplicated studies. Discrepancies were resolved by mutual consent obtained from another author (D.B.S.). A Microsoft Excel sheet (Microsoft Corp) was used to extract the study characteristics, such as type of study, year of publication, country, number of CTH studies, number of patients with AMS, number of CTHs in patients with AMS, CTH study outcome, cost of CTH study, and change in management.

### Outcome Measures

The primary outcome was the proportion (event rate) of CTH use in patients with acute atraumatic AMS. Secondary outcomes were (1) the proportion (event rate) of positive CTH results; (2) outcomes of CTH; (3) use of CTH in the ED, ICU, or inpatient unit; (4) change in management per CTH result; and (5) cost of CTH study. We also performed a pooled proportion meta-analysis comparing the US and European studies of CTH event rates.

### Evidence of Study Quality

The Joanna Briggs Institute’s critical appraisal checklist for cohort, case-control, and quasiexperimental studies was used to evaluate the quality of the studies.^8^ The checklist included 11 to 13 items; if the answer to an item was yes, the study was scored 1; otherwise it was scored zero. Total quality scores of 4 or less, 5 to 7, and 8 or greater were considered low, moderate, and high quality, respectively (eTables 2-4 in the Supplement).^9^

### Statistical Analysis

The aggregate proportion of CTH prevalence (event rate) in patients with AMS and positive CTH findings was pooled using Stata software, version 17.0 (StataCorp LLC). Proportions were used to estimate the outcomes with a 95% CI. We assumed significant heterogeneity among the studies; hence, the random-effects model was used for meta-analysis. The between-study heterogeneities were assessed, with *P* < .10 or *I*^2^ > 50% indicating significant heterogeneity. In addition, sensitivity analyses were conducted to test the stability of the pooled proportions by excluding studies one by one. Finally, publication bias was assessed with a funnel plot.

## Results

### Literature Search

**Figure 1.**
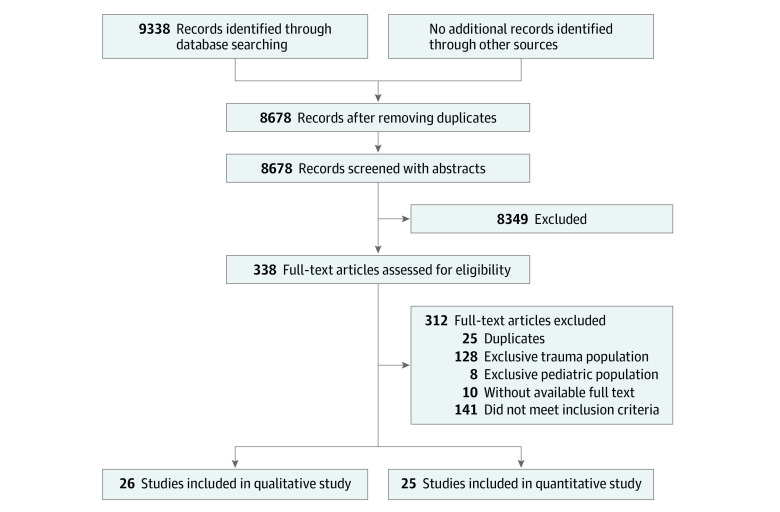
PRISMA Diagram of Eligible Studies

A total of 9338 studies were identified, and no additional records were obtained from other sources. Six hundred fifty-one studies were duplicated and omitted. A total of 8687 studies were screened with title and abstract, of which 338 qualified for full-text review. After applying inclusion and exclusion criteria, 26 were qualified for systematic review, and 25 were eligible for meta-analysis, with a combined total of 79 201 patients (Figure 1).

### Study Characteristics

Of 26 studies, 11 were performed in the ED,^5,10,11,12,13,14,15,16,17,18,19^ 3 in the ICU,^20,21,22^ 4 in an inpatient unit,^23,24,25,26^ and 8 in mixed settings.^27,28,29,30,31,32,33,34^ Fifteen studies were performed in the US,^5,10,11,18,20,22,24,25,26,27,28,29,30,33,34^ and 4 in Europe^13,16,21,31^ (Table). The studies were appraised using the Joanna Briggs Institute quality assessment. We rated 11 studies as high quality^12,16,19,22,23,27,28,31,32,33,34^ and 15 studies as medium quality^5,10,11,13,14,15,17,18,20,21,24,25,26,29,30^; none of the studies were rated as low quality (eTables 2-4 in the Supplement).

**Table.** Descriptive Analysis of Studies^a^

Source	Type of study	Place of study	Total No. of patients/No. of patients with AMS	No. of CTH events in patients with AMS	No. of positive CTH events	Setting	Outcome of CTH (defined in study)	Outcome of positive CTH findings (defined in patients with AMS)	Time to CTH	Change in management
Bent et al,^10^ 2015	Retrospective, cohort	US	500/65	65	7	ED	Acute/subacute stroke, ICH, mass, edema, or mass effect	NA	NA	NA
Callen et al,^11^ 2020	Retrospective, case-control	US	9593/1816	1816	130	ED	Hemorrhage, hydrocephalus, mass effect, or worsening of prior finding	NA	NA	NA
Chen et al,^12^ 2020	Retrospective, case-control	Taiwan	66/66	31	0	ED	Intracerebral hemorrhage, new ischemic infarction, or space-occupying lesions	None	12 h	None
Chokshi et al,^20^ 2016	Retrospective, cohort	US	2846/2846	2846	566	ICU	Hemorrhage, infarction, mass effect, or hydrocephalus	NA	90 min	NA
Covino et al,^13^ 2019	Observational, quasi-experimental	Italy	1664/574	574	NA (not reported for AMS)	ED	Ischemia, bleeding, or hydrocephalus	NA	NA	NA
Detweiler et al,^27^ 2020	Retrospective, case-control	US	200/100	79	NA	Mixed	White matter lesion	NA	NA	NA
Detweiler et al,^28^ 2017^b^	Retrospective, case-control	US	200/100	79	NA	Mixed	White matter lesion, cerebral atrophy, or intracerebral calcifications	NA	NA	NA
Donovan et al,^29^ 2015	Retrospective, cohort	US	462/ 302	302	1	Mixed	ICH	Yes (ICH)	NA	Yes, intubated
Finkelmeier et al,^21^ 2019	Retrospective, case-control	Germany	690/162	162	16	ICU	ICH/SAH or stroke	NA	NA	Yes, but not clear
Hanna et al,^30^ 2021	Retrospective, cohort	US	520/408	408	3	Mixed	Acute ischemic stroke, hemorrhagic stroke, or SDH	NA	NA	NA
Hufschmidt et al,^31^ 2008	Retrospective, cohort	Germany	294/294	178	25	Mixed	Diagnostic to the acute state (no clear mention of diagnosis)	NA	NA	NA
Khan et al,^22^ 2014	Retrospective, cohort	US	901/635	635	47	ICU	Acute and subacute or chronic changes (infarction, hemorrhage, mass, or hydrocephalus)	NA	NA	NA
Lai et al,^23^ 2010	Retrospective, case-control	Australia	200/25	25	6	Inpatient	Acute ischemic stroke, hemorrhagic stroke, or SDH	NA	NA	NA
Lim et al,^32^ 2009	Retrospective, cohort	Singapore	578/578	327	128	Mixed	Acute infarct or ICH	NA	NA	NA
Nesselroth et al,^14^ 2021	Retrospective, cohort	Israel	1536/116	116	23	ED	Mass effect, herniation, ischemic stroke, or ICH	NA	NA	NA
Patel et al,^33^ 2002	Retrospective, case-control	US	152/151	42	0	Mixed	Acute, chronic, and none	NA	12 h	None
Patel et al,^15^ 2019	Retrospective, cohort	India	308/55	55	17	ED	Infarct, ICH, or mass	Yes (SCH, 9/17)	NA	NA
Rahimi et al,^34^ 2016	Retrospective, case-control	US	349/349	223	25	Mixed	CVA, ICH, or mass	Yes (CVA, 13/25; ICH, 9/25)	NA	NA
Segard et al,^16^ 2013	Retrospective, case-control	France	291/139	139	63	ED	Stroke, TIA, tumors, and ICH	NA	NA	NA
Shuaib et al,^18^ 2014	Retrospective, cohort	US	379/49	49	12	ED	Mass, ischemia, ICH, or calcifications	NA	NA	NA
Sinclair et al,^17^ 1993	Retrospective, cohort	Canada	416/25	25	8	ED	Pathology not previously demonstrated (infarction, bleed, or SAH)	NA (not clear if outcome mentioned for AMS)	NA	Yes (surgery)
Thacker et al,^24^ 2021	Retrospective, cohort	US	83/58	58	1	Inpatient	SDH	Yes (SDH)	NA	Yes (surgery)
Theisen-Toupal et al,^25^ 2014	Retrospective, cohort	US	220/220	220	6	Inpatient	Stroke/ICH	NA	NA	Yes
Tu et al,^5^ 2022	Retrospective, cohort	US	52 799/28 332	6146	588	ED	Critical results (ischemic infarct, ICH, or mass lesion)	NA	NA	NA
Wang and You,^19^ 2013	Retrospective, cohort	Canada	3967/1120	1120	NA	ED	Ischemia, hemorrhage, or mass needing follow-up	NA	NA	NA
Wong et al,^26^ 2014	Retrospective, cohort	US	187/139	126	0	Inpatient	Ischemia, hemorrhage, or acute organic disorder	None	NA	NA

Abbreviations: AMS, altered mental status; CTH, computed tomography of head; CVA, cerebrovascular accident; ED, emergency department; ICH, intracranial hemorrhage; ICU, intensive care unit; NA, not available; SAH, subarachnoid hemorrhage; SDH, subdural hematoma; TIA, transient ischemic attack.

^a^ If there was no explicit mention of contrast-enhanced CT used in the study, noncontrast CTH was assumed. If there was no explicit mention of the study setting, mixed setting (ED, inpatient unit, or ICU) was assumed. If there was no explicit mention of focal neurologic deficit in the study, it was assumed focal neurologic deficit was not associated with AMS. Therefore, the studies with AMS and focal neurologic deficit mentioned that did not allow the authors to segregate the patients with AMS were excluded.

^b^ Study not included in the meta-analysis because the same patient sample was used in another study by the authors.

### Outcome Analyses

#### Primary Outcome

**Figure 2.**
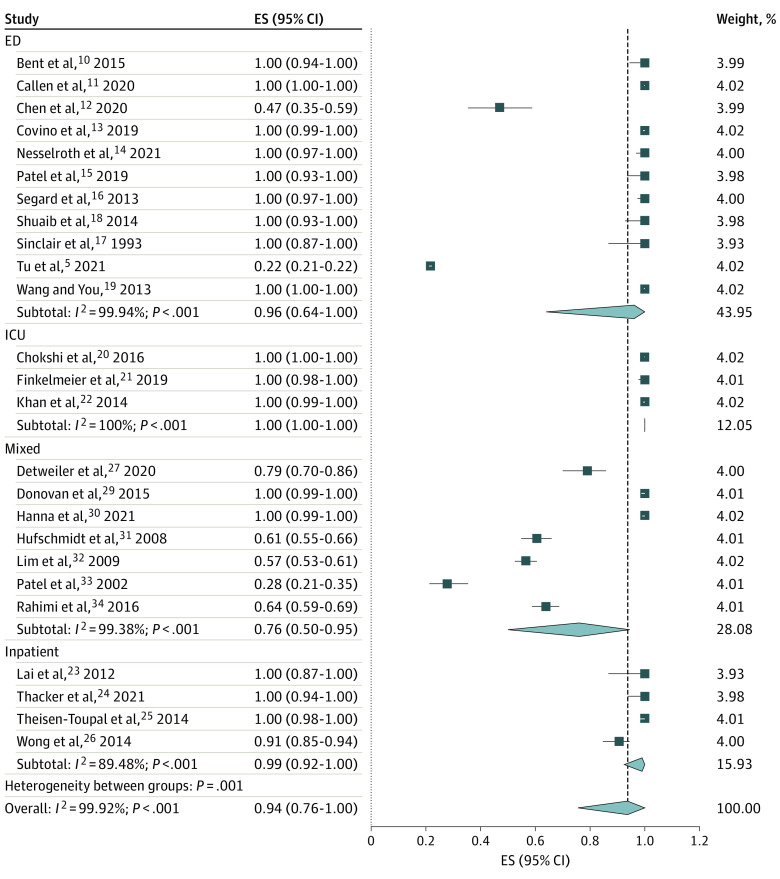
Proportion of Computed Tomography of Head in Patients With Altered Mental Status Among Studies Effect size (ES) represents the proportion of computed tomography of the head in patients with altered mental states. The model used is the random-effects model. ED indicates emergency department; ICU, intensive care unit.

The CTH event rate among patients with AMS was 94% (proportion, 0.94; 95% CI, 0.76-1.00). Additional subgrouping for CTH event based on the settings showed 100% in the ICU (proportion, 1.00; 95% CI, 1.00-1.00), followed by 99% in the inpatient unit (proportion, 0.99; 95% CI, 0.92-1.00), 96% in the ED (proportion, 0.96; 95% CI, 0.64-1.00), and 76% among mixed settings (proportion, 0.76; 95% CI, 0.50-0.95) (Figure 2). In the event rate, total CTH performed on patients with AMS was calculated. For example, if a study had a patient who had 2 CTH studies performed for AMS and reported, we counted the CT performed as 2 CTH studies.

#### Secondary Outcomes

**Figure 3.**
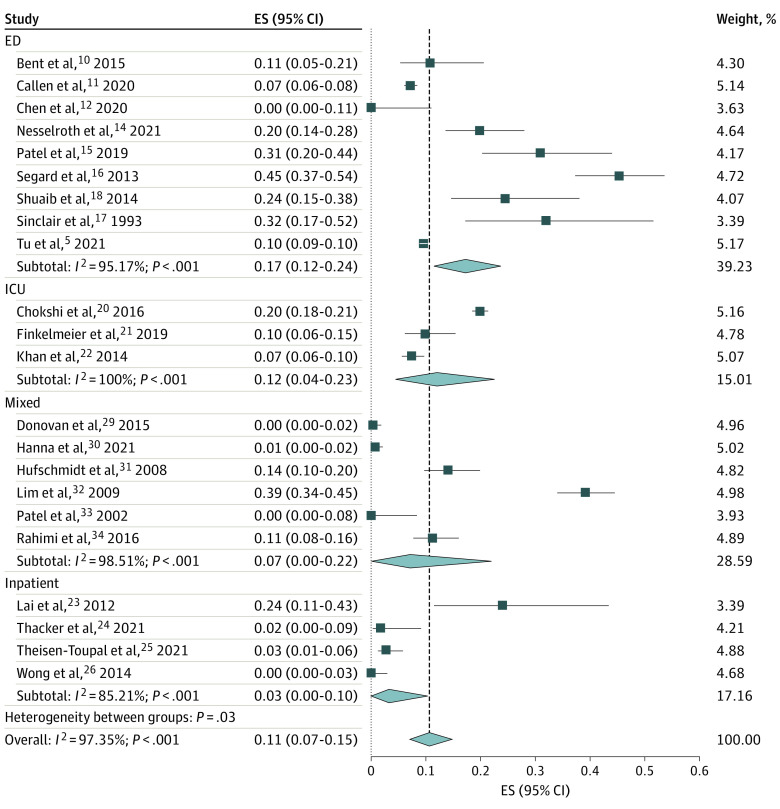
Proportion of Positive Computed Tomography of Head Findings in Patients With Altered Mental Status Among Studies Effect size (ES) represents the proportion of positive findings on computed tomography of the head in patients with altered mental states. The model used is the random-effects model. ED indicates emergency department; ICU, intensive care unit.

The overall positive CTH event rate within those CTH studies was 11% (proportion, 0.11; 95% CI, 0.07-0.15), with a maximum of 17% (proportion, 0.17; 95% CI, 0.12-0.24) in the ED, followed by 12% in the ICU (proportion, 0.12; 95% CI, 0.04-0.23), 7% in mixed settings (proportion, 0.07; 95% CI, 0.00-0.22), and 3% in the inpatient setting (proportion, 0.03; 95% CI, 0.00-0.10) (Figure 3). In both models, significant heterogeneity was found among the analyzed studies (*I*^2^ > 50%) (Figure 2 and Figure 3). Only 2 studies discussed the time taken to perform a CTH study, with 12 hours^12,33^ and 90 minutes^20^ being reported. Furthermore, only 1 article^18^ discussed the cost related to CTH study. Although all studies included declared the definition of positive CTH findings in the study, only 4 discussed the details of CTH results pertinent to patients with AMS.^15,24,29,34^ Four studies mentioned a change in management based on CTH results.^17,21,24,29^ The CTH event rates were 92% (proportion, 0.92; 95% CI, 0.61-1.00) in the US and 96% (proportion, 0.96; 95% CI, 0.74-1.00) in Europe (eFigure 1 in the Supplement).

### Publication Bias

Publication bias was assessed using funnel plots. For both the primary and secondary outcomes evaluated, funnel plots showed a significantly asymmetric distribution of studies, suggesting significant publication bias across studies (eFigures 2-3 in the Supplement).

### Sensitivity Analysis

After removing each study individually and calculating the proportions, the results did not fluctuate. The CTH event rates ranged from 85% to 88% (*P* < .001), and positive CTH event rates ranged from 11% to 14% (*P* < .001) (eFigures 4-5 in the Supplement). This finding demonstrates that our results are reliable.

## Discussion

In this meta-analysis, we report the proportion of noncontrast CTH use among patients presenting with atraumatic acute AMS. The use of CTH was 94%, and a positive result was found among 11% of the CTH studies performed. We included 26 studies in the systematic review and excluded 1 study in the meta-analysis because the authors used the same set of participants in another study.

Of the studies that met the inclusion and exclusion criteria and were included in this study, 17 were retrospective cohort studies and 9 were retrospective case-control studies. The majority of the studies were on CTH use in different clinical settings, and patients with AMS were a subset. We performed a pooled analysis of the event rate in terms of CTH use in the acute AMS setting and the event rate of positive CTH findings among those CTH studies performed. Because of the inclusion of such variable studies, we had high heterogeneity in the pooled analysis (CTH event rate: *I*^2^ = 99.92%, *P* < .001; positive CTH event rate: *I*^2^ = 97.35%, *P* < .001). However, because this study aimed to calculate the event rate, we expected significant heterogeneity among the studies.

The main reasons for performing CTH studies in clinical practice on patients with AMS are the lack of clear guidelines,^4^ diagnostic dilemmas, and defensive medicine.^2,3^ However, in the subgroup analysis of the studies in the US and Europe, the use of CTH in Europe was not different from that in the US despite socialized medicine and presumed fewer medical-legal concerns. Tu et al^5^ reported that AMS accounted for the highest frequency of CTH ordered in the ED in a single health system study; 46% of CTH studies were ordered for AMS alone, with a 10% yield. Computed tomography of the head comprised 38% of all CT studies ordered in the ED and 8% of total ED visits.^5^ Our subgroup analysis found that the CTH event rate was 96% with a positive CTH event rate of 17% in the ED. A few studies suggested that the use of imaging in the ED setting was associated with increased length of stay, higher bed occupancy, and increased cost of health care.^3,35,36^ There is wide variation in price transparency and cost of medical imaging, and the reported cost of a CTH study can range from $211 to $2200.^36^ However, in our systematic review, we found only 1 study that discussed the cost of CTH studies.^18^

Computed tomographic studies are associated with significant radiation exposure and cancer risk. A single CT study’s mean radiation dose is typically 15 mSv in adults and 30 mSv in neonates. A significant overall risk of cancer was found in nuclear bomb survivors who had radiation exposure ranging from 5 to 150 mSv (mean, 40 mSv). This radiation dose is equivalent to the relevant organ dose received with 2 or 3 scans in an adult from a typical CT study.^2^ For CTH, even with newer CT machines, radiation ranges from 1 to 10 mSv per CTH in an adult,^4^ which means a person who received 3 to 4 CT studies in a lifetime has a similar risk of cancer as those who survived nuclear bombs in Japan. Although the cancer risk estimates calculated from a single CT study appear to be highest at the age when the CT was performed and decrease after that,^2^ so far, no clinical study has compared the lifetime vs short-period risks of cancer pertaining to CT studies. The linear no-threshold (LNT) concept advocates that even a small dose of radiation increases genetic alterations, which increases cancer risks.^37^ Some animal, cellular, and molecular studies demonstrated that the responses at the cellular level after repeated low-dose exposures were less pronounced than the total dose delivered at a high dose.^38^ Recently, there has been debate around the LNT concept because some epidemiologic studies showed that radiation doses less than 100 mSv may not be enough to induce carcinogenesis.^38,39^ The LNT model is criticized by some for not being biologically plausible, overestimating radiation risks, and preventing necessary imaging, causing more harm than benefit.^37,38,39^ Some even advocate for low-dose radiation for its presumed benefit, known as the radiation hormesis concept.^37^ Regardless of the criticism, the LNT model has been adopted by national and international advisory bodies and has guided radiation protection policies for decades.^37^ There is no argument regarding the wise use of imaging studies, such as CT, based on risk-benefit ratio assumptions.^39^

Health care professionals’ awareness regarding radiation hazards associated with CT studies is alarmingly low. In a survey study, 53% of radiologists and 91% of ED physicians did not believe that CT studies can increase the lifetime risk of cancer.^6^ Moreover, another study suggested that approximately one-third of the CT studies could be replaced by alternative approaches or no study at all.^40^ Given the risk of radiation hazards and the added cost of care, clinicians should use CTH judiciously. Prospective studies involving a larger cohort of patients with acute change in mental status are required to develop clinical risk stratification tools to facilitate rational and judicious use of CTH. Our results align with the recommendations of the Choosing Wisely and Image Wisely campaigns that advocate against the overuse of CTH.^41^ In addition, professional and societal bodies should use awareness campaigns considering the limited awareness among health care professionals regarding radiation hazards related to CT studies.

### Limitations

Our study had some limitations. The first is heterogeneity. The studies included had a wide variation in sample size and settings, which introduced significant heterogeneity in the study. However, this variation is a common occurrence with meta-analyses for incidence or event rate. Second, in the outcome analysis, we could not report the usefulness of positive CTH results. Only 4 studies discussed the diagnoses of positive CTH findings, and none of the studies explicitly discussed the change of management from the CTH results. Third, although the quality of the studies was moderate to high, the studies had low levels of evidence. Fourth, we had a significantly asymmetric funnel plot that explains the study’s publication bias, but we suspect this asymmetry is partly attributable to heterogeneity.^42^ Furthermore, the sensitivity analysis supported the reliability of the results. Regardless of these limitations, this is the first systematic review and meta-analysis, to our knowledge, that investigated the use of noncontrast CTH among patients with acute atraumatic AMS.

## Conclusions

This meta-analysis found that the use of noncontrast CTH in patients with acute-onset atraumatic AMS is high, with a low yield for a positive result. Computed tomographic studies are associated with significant radiation exposure. Future large-scale studies are required to provide more reliable estimates for the diagnostic yield of CTH among patients with AMS. Clinicians should exercise caution and use their clinical judgment to minimize the indiscriminate use of CTH in the evaluation of patients with AMS.

## Notes

Supplement. **eTable 1.** Database Search Strategy **eTable 2.** The Joanna Briggs Institute’s (JBI) Critical Appraisal Checklist for Case-Control Study **eTable 3.** The Joanna Briggs Institute’s (JBI) Critical Appraisal Checklist for Cohort Study **eTable 4.** The Joanna Briggs Institute’s (JBI) Critical Appraisal Checklist for Quasi-Experimental Study **eFigure 1.** The Proportion of Computed Tomography of the Head (CTH) in Patients With Altered Mental Status (AMS) Among Studies **eFigure 2.** Funnel Plot Showing the Asymmetric Distribution of Studies Suggesting Significant Publication Bias for Computed Tomography of the Head (CTH) Events **eFigure 3.** Funnel Plot Showing the Asymmetric Distribution of Studies Suggesting Significant Publication Bias for Positive Computed Tomography of the Head (CTH) Events **eFigure 4.** Sensitivity Analysis of Studies for Computed Tomography of the Head (CTH) Events **eFigure 5.** Sensitivity Analysis of Studies for Positive Computed Tomography of the Head (CTH) Events
