# Supplementary material for: Use of Computed Tomography of the Head in Patients With Acute Atraumatic Altered Mental Status: A Systematic Review and Meta-analysis
Source: JAMA Netw Open. 2022 Nov 18;5(11):e2242805. doi: 10.1001/jamanetworkopen.2022.42805 (PMC9675006; doi:10.1001/jamanetworkopen.2022.42805)
Supplement: Supplement. — eTable 1. Database Search Strategy eTable 2. The Joanna Briggs Institute’s (JBI) Critical Appraisal Checklist for Case-Control Study eTable 3. The Joanna Briggs Institute’s (JBI) Critical Appraisal Checklist for Cohort Study eTable 4. The Joanna Briggs Institute’s (JBI) Critical Appraisal Checklist for Quasi-Experimental Study eFigure 1. The Proportion of Computed Tomography of the Head (CTH) in Patients With Altered Mental Status (AMS) Among Studies eFigure 2. Funnel Plot Showing the Asymmetric Distribution of Studies Suggesting Significant Publication Bias for Computed Tomography of the Head (CTH) Events eFigure 3. Funnel Plot Showing the Asymmetric Distribution of Studies Suggesting Significant Publication Bias for Positive Computed Tomography of the Head (CTH) Events eFigure 4. Sensitivity Analysis of Studies for Computed Tomography of the Head (CTH) Events eFigure 5. Sensitivity Analysis of Studies for Positive Computed Tomography of the Head (CTH) Events [file jamanetwopen-e2242805-s001.pdf]

# Supplementary Online Content

Acharya R, Kafle S, Shrestha DB, et al. Use of computed tomography of the head in patients with acute atraumatic altered mental status: a systematic review and meta-analysis. *JAMA Netw Open*. 2022;5(11):e2242805. doi:10.1001/jamanetworkopen.2022.42805

**eTable 1.** Database Search Strategy
**eTable 2.** The Joanna Briggs Institute's (JBI) Critical Appraisal Checklist for Case-Control Study
**eTable 3.** The Joanna Briggs Institute's (JBI) Critical Appraisal Checklist for Cohort Study
**eTable 4.** The Joanna Briggs Institute's (JBI) Critical Appraisal Checklist for Quasi-Experimental Study
**eFigure 1.** The Proportion of Computed Tomography of the Head (CTH) in Patients With Altered Mental Status (AMS) Among Studies
**eFigure 2.** Funnel Plot Showing the Asymmetric Distribution of Studies Suggesting Significant Publication Bias for Computed Tomography of the Head (CTH) Events
**eFigure 3.** Funnel Plot Showing the Asymmetric Distribution of Studies Suggesting Significant Publication Bias for Positive Computed Tomography of the Head (CTH) Events
**eFigure 4.** Sensitivity Analysis of Studies for Computed Tomography of the Head (CTH) Events
**eFigure 5.** Sensitivity Analysis of Studies for Positive Computed Tomography of the Head (CTH) Events

This supplementary material has been provided by the authors to give readers additional information about their work.

© 2022 Acharya R et al. *JAMA Network Open.*

**eTable 1.** Database Search Strategy

| |
|---|
| **Building block approach to search database**<br><br>Altered mental status: "altered mental status"[tw] OR confusion[tw] OR disorientation OR unconscious*[tw] OR AMS[tw]<br>CT head: "Tomography, X-ray Computed"[Mesh] OR "CT Head" OR CTH |
| **PubMed/MEDLINE, PubMed Central**<br>("altered mental status"[tw] OR confusion[tw] OR disorientation OR unconscious*[tw] OR AMS[tw]) AND ("Tomography, X-Ray Computed"[Mesh] OR "CT Head"[tw] OR CTH[tw])<br><br>**PubMed/Medline: 2308 Hits**<br>https://pubmed.ncbi.nlm.nih.gov/?term=%28%22altered+mental+status%22%5Btw%5D+OR+confusion%5Btw%5D+OR+disorientation+OR+unconscious*%5Btw%5D+OR+AMS%5Btw%5D%29+AND+%28%22Tomography%2C+X-Ray+Computed%22%5BMesh%5D+OR+%22CT+Head%22%5Btw%5D+OR+CTH%5Btw%5D%29&size=200<br>**PubMed Central: 4671 Hits**<br>https://www.ncbi.nlm.nih.gov/pmc/?term=(%22altered+mental+status%22%5Btw%5D+OR+confusion%5Btw%5D+OR+disorientation+OR+unconscious*%5Btw%5D+OR+AMS%5Btw%5D)+AND+(%22Tomography%2C+X-Ray+Computed%22%5BMesh%5D+OR+%22CT+Head%22%5Btw%5D+OR+CTH%5Btw%5D) |
| **CINAHL: 628 Hits**<br>("altered mental status" OR confusion OR delirium OR disorientation OR unconscious OR AMS) AND ("Tomography, X-Ray Computed" OR "CT Head" OR CTH)<br>https://web-p-ebscohost-com.proxy.campbell.edu/ehost/results?vid=1&sid=315f5b5a-d468-4b0d-9b3c-ca1abed29ccb%40redis&bquery=(%22altered+mental+status%22+OR+confusion+OR+delirium+OR+disorientation+OR+unconscious+OR+AMS)+AND+(%22Tomography%2c+X-Ray+Computed%22+OR+%22CT+Head%22+OR+CTH)&bdata=JmRiPWNjbSZ0eXBlPTAmc2VhcmNoTW9kZT1TdGFuZGFyZCZzaXRlPWVob3N0LWxpdmU%3d |
| **EMBASE: 1731 hits**<br>('altered mental status'/exp OR 'altered mental status' OR 'confusion'/exp OR confusion OR 'delirium'/exp OR delirium OR 'disorientation'/exp OR disorientation OR 'unconscious'/exp OR unconscious OR ams) AND ('tomography, x-ray computed'/exp OR 'tomography, x-ray computed' OR 'ct head' OR cth)<br>https://www.embase.com/?phase=continueToApp#advancedSearch/resultspage/history.1/page.1/25.items/orderby.date/source. |

© 2022 Acharya R et al. *JAMA Network Open.*

**eTable 2.** The Joanna Briggs Institute's (JBI) critical appraisal checklist for case-control study. Total quality scores ≤ 4, 5 to 7, and ≥ 8 were considered as low, moderate and high quality respectively

| Study | Were the groups comparable other than the presence of disease in cases or the absence of disease in controls? | Were cases and controls matched appropriately? | Were the same criteria used for identification of cases and controls? | Was exposure measured in a standard, valid and reliable way? | Was exposure measured in the same way for cases and controls? | Were confounding factors identified? | Were strategies to deal with confounding factors stated? | Were outcomes assessed in a standard, valid and reliable way for cases and controls? | Was the exposure period of interest long enough to be meaningful? | Was appropriate statistical analysis used? | Score | Quality |
|---|---|---|---|---|---|---|---|---|---|---|---|---|
| Callen A et al. (2020) | yes | n/a | yes | yes | yes | n/a | n/a | yes | yes | yes | 7 | moderate |
| Chen H et al. (2020) | yes | Yes | yes | yes | yes | yes | yes | yes | yes | yes | 10 | high |
| Detweiler M et al. (2020) | yes | Yes | yes | yes | yes | yes | no | yes | yes | yes | 9 | high |
| Detweiler M et al. (2017) | yes | Yes | yes | yes | yes | yes | no | yes | yes | yes | 9 | high |
| Finkelmeier F et al. (2019) | no | n/a | yes | yes | yes | unclear | yes | yes | yes | yes | 7 | moderate |
| Lai M et al. (2010) | yes | No | yes | yes | yes | yes | yes | yes | yes | yes | 9 | high |
| Patel M et al. (2012) | yes | Yes | yes | yes | yes | unclear | yes | yes | yes | yes | 8 | high |
| Rahimi R et al. (2016) | yes | Yes | yes | yes | yes | unclear | yes | yes | yes | yes | 9 | high |
| Segard J et al. (2013) | no | Yes | yes | yes | yes | unclear | yes | yes | yes | yes | 8 | high |

© 2022 Acharya R et al. *JAMA Network Open.*

**eTable 3.** The Joanna Briggs Institute's (JBI) critical appraisal checklist for cohort study. Total quality scores ≤ 4, 5 to 7, and ≥ 8 were considered as low, moderate and high quality respectively.

| **Study** | **Were the two groups similar and recruited from the same population?** | **Were the exposures measured similarly to assign people to both exposed and unexposed groups?** | **Was the exposure measured in a valid and reliable way?** | **Were confounding factors identified?** | **Were strategies to deal with confounding factors stated?** | **Were the groups /participants free of the outcome at the start of the study (or at the moment of exposure)?** | **Were the outcomes measured in a valid and reliable way?** | **Was the follow up time reported and sufficient to be long enough for outcomes to occur?** | **Was follow up complete, and if not, were the reasons to loss to follow up described and explored?** | **Were strategies to address incomplete follow up utilized?** | **Was appropriate statistical analysis used?** | **Score** | **Quality** |
|---|---|---|---|---|---|---|---|---|---|---|---|---|---|
| Bent C et al. (2015) | n/a | n/a | yes | yes | no | yes | yes | yes | yes | n/a | yes | 7 | moderate |
| Chokshi F et al. (2016) | n/a | n/a | yes | no | n/a | yes | yes | yes | yes | n/a | yes | 6 | moderate |
| Donovan L et al. (2015) | yes | n/a | yes | no | n/a | yes | yes | yes | yes | n/a | yes | 7 | moderate |
| Hanna A et al (2021) | n/a | n/a | yes | yes | yes | yes | yes | yes | yes | n/a | yes | 7 | moderate |
| Hufschmidt A et al. (2008) | yes | yes | yes | unclear | unclear | yes | yes | yes | yes | n/a | yes | 8 | high |
| Khan S et al. (2014) | yes | yes | yes | yes | yes | yes | yes | yes | yes | n/a | yes | 10 | high |
| Lim BL et al. (2009) | yes | yes | yes | unclear | unclear | yes | yes | yes | yes | n/a | yes | 8 | high |
| Nesselroth D et al. (2021) | n/a | n/a | yes | n/a | n/a | yes | yes | yes | yes | n/a | yes | 6 | moderate |
| Patel R et al. (2019) | n/a | n/a | yes | n/a | n/a | yes | yes | yes | yes | n/a | yes | 6 | moderate |
| Shuaib W et al (2014) | n/a | n/a | yes | n/a | n/a | yes | yes | yes | yes | n/a | yes | 6 | moderate |

| | | | | | | | | | | | | | |
|---|---|---|---|---|---|---|---|---|---|---|---|---|---|
| Sinclair D et al. (1993) | n/a | n/a | yes | n/a | n/a | yes | yes | yes | yes | n/a | yes | 6 | moderate |
| Thacker P et al. (2021) | n/a | n/a | yes | n/a | n/a | yes | yes | yes | yes | n/a | yes | 6 | moderate |
| Theisen-Toupal J et al. (2014) | n/a | n/a | yes | unclear | unclear | yes | yes | yes | yes | n/a | yes | 6 | moderate |
| Tu L et al (2021) | n/a | n/a | yes | n/a | n/a | yes | yes | yes | yes | n/a | yes | 5 | moderate |
| Wang X et al. (2013) | yes | yes | yes | unclear | yes | yes | yes | yes | yes | n/a | yes | 9 | high |
| Wong J et al. (2014) | yes | yes | yes | no | n/a | yes | yes | yes | yes | n/a | yes | 8 | moderate |

© 2022 Acharya R et al. *JAMA Network Open.*

**eTable 4.** The Joanna Briggs Institute's (JBI) critical appraisal checklist for quasi-experimental study. Total quality scores ≤ 4, 5 to 7 and ≥ 8 were regarded as low, moderate, and high quality, respectively.

| Study | Is it clear in the study what is the 'cause' and what is the 'effect' (i.e. there is no confusion about which variable comes first)? | Were the participants included in any comparisons similar? | Were the participants included in any comparisons receiving similar treatment/care, other than the exposure or intervention of interest? | Was there a control group? | Were there multiple measurements of the outcome both pre and post the intervention/exposure? | Was follow up complete and if not, were differences between groups in terms of their follow up adequately described and analyzed? | Were the outcomes of participants included in any comparisons measured in the same way? | Were outcomes measured in a reliable way? | Was appropriate statistical analysis used? | Score | Quality |
|---|---|---|---|---|---|---|---|---|---|---|---|
| Covino M eta l. (2019) | yes | yes | n/a | n/a | yes | Yes | yes | yes | yes | 7 | moderate |

© 2022 Acharya R et al. *JAMA Network Open.*

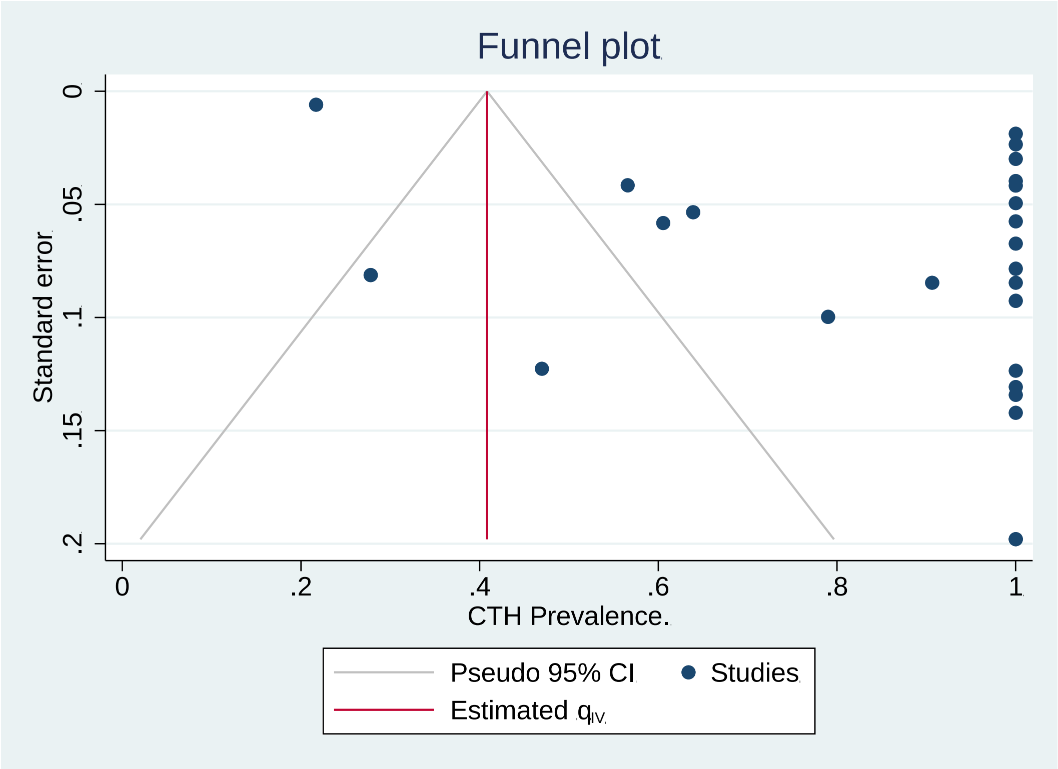


**eFigure 1.** Funnel Plot Showing the Asymmetric Distribution of Studies Suggesting Significant Publication Bias for Computerized Tomography of Head (CTH) Events

© 2022 Acharya R et al. *JAMA Network Open.*

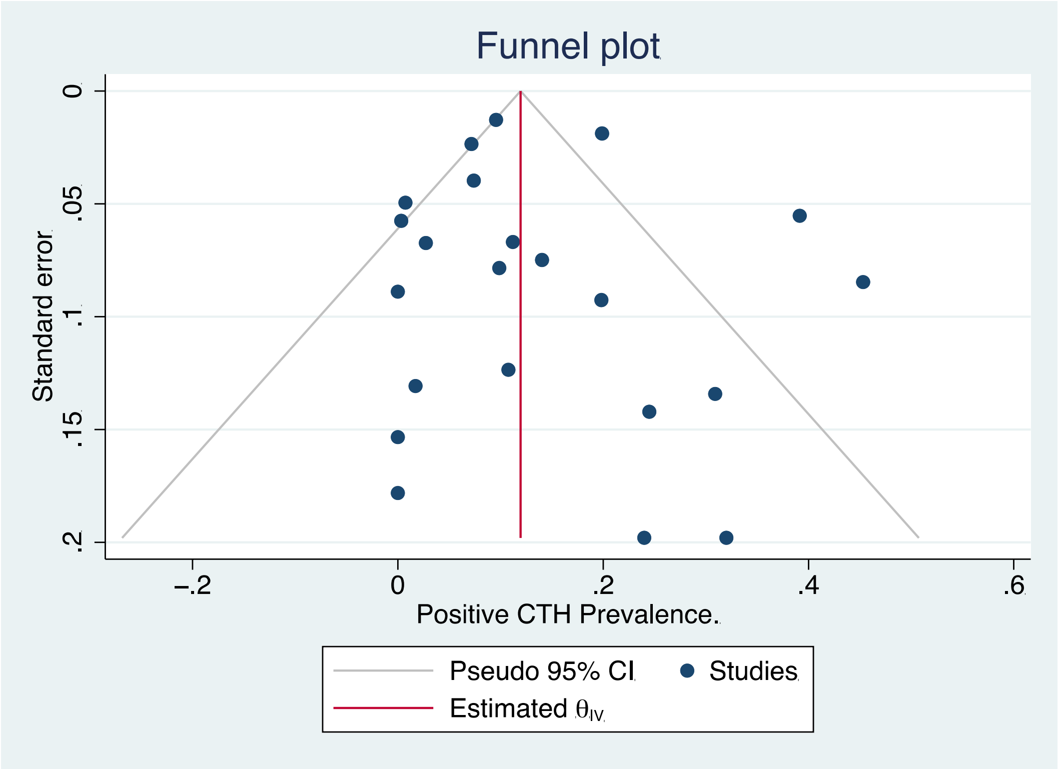


**eFigure 2.** Funnel Plot Showing the Asymmetric Distribution of Studies Suggesting Significant Publication Bias for Positive Computerized Tomography of Head (CTH) Events

© 2022 Acharya R et al. *JAMA Network Open.*

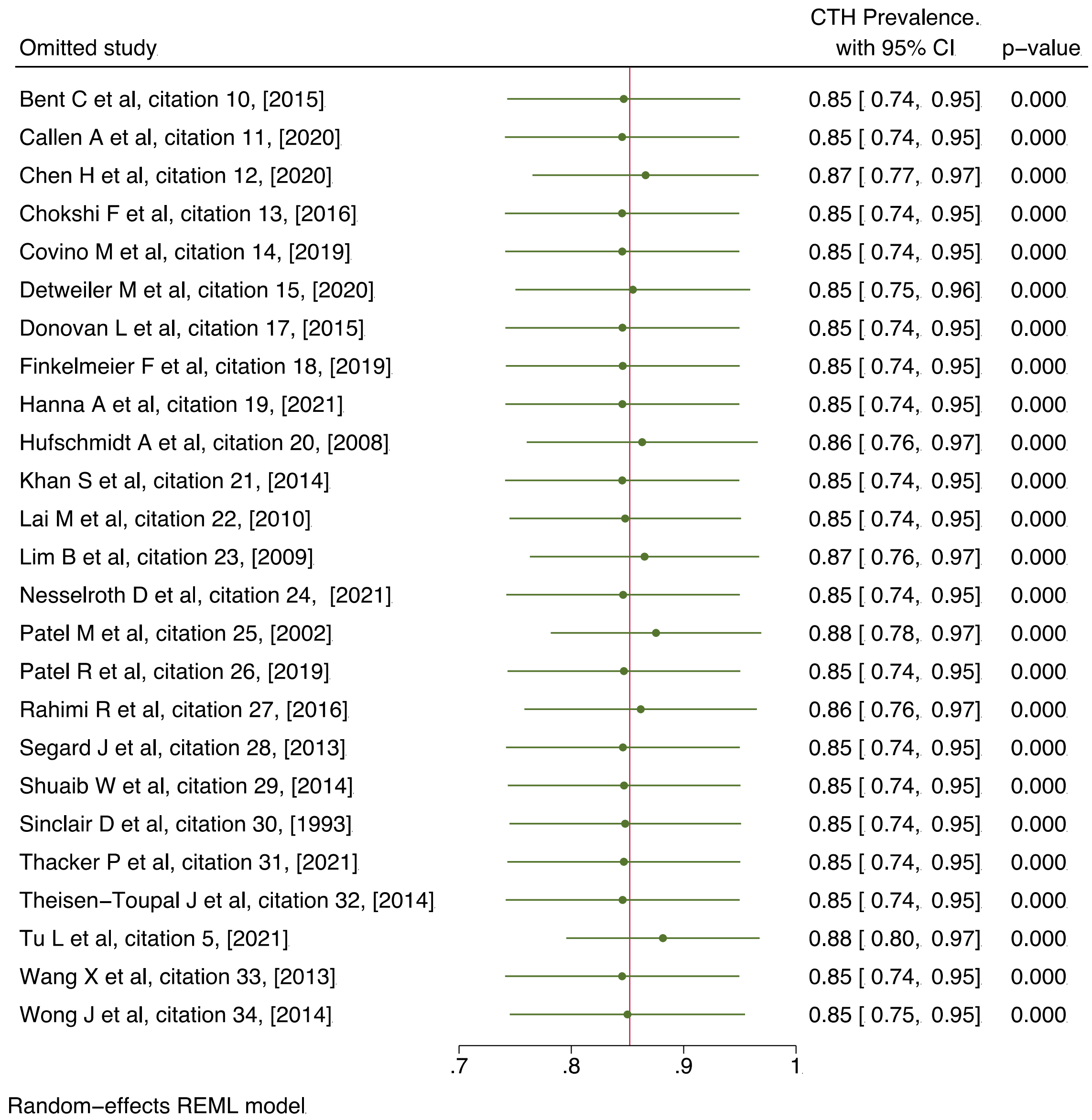


**eFigure 3.** Sensitivity Analysis of Studies for Computerized Tomography of Head (CTH) Event

© 2022 Acharya R et al. *JAMA Network Open.*

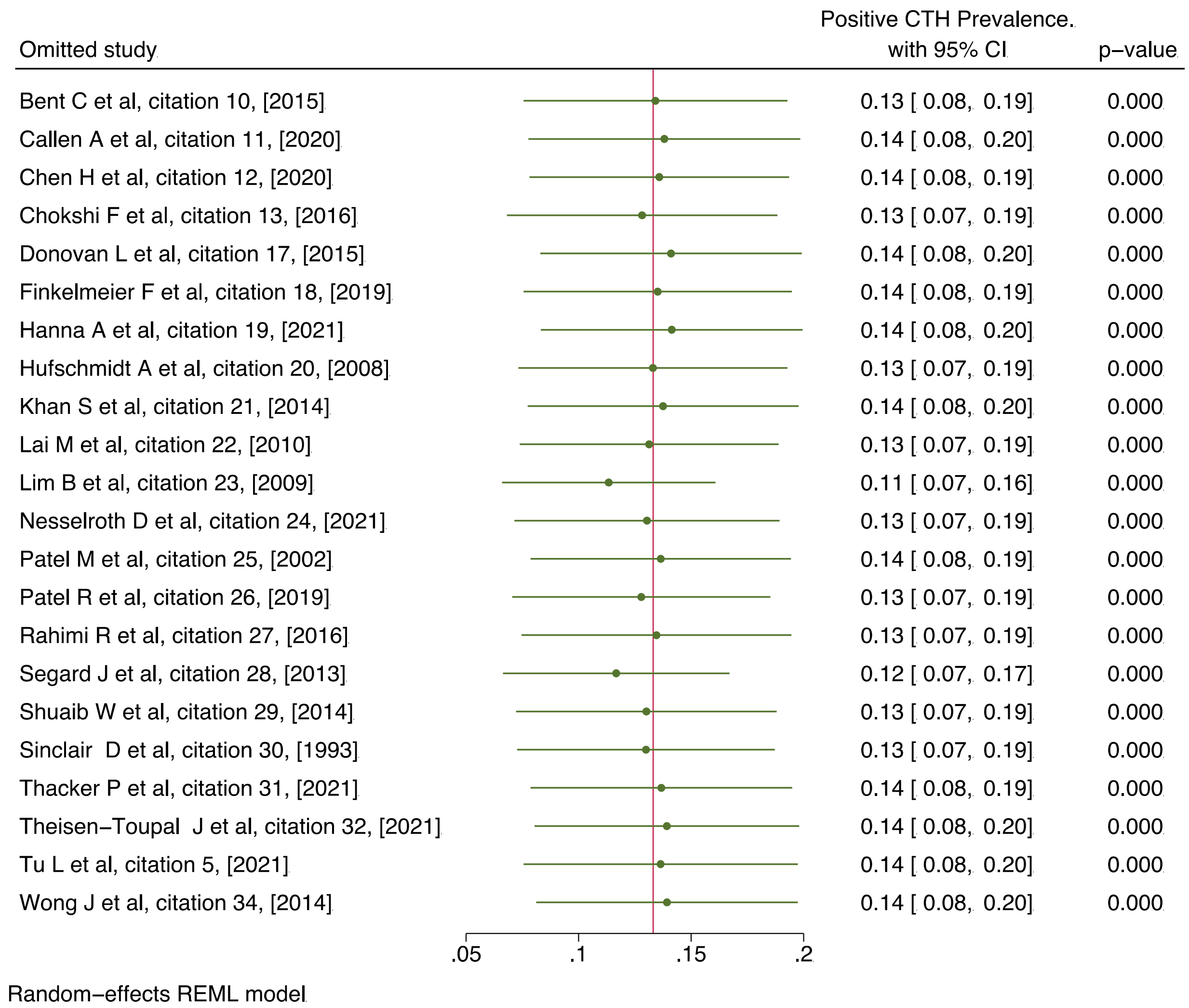


**eFigure 4.** Sensitivity Analysis of Studies for Positive Computerized Tomography of Head (CTH) Events

© 2022 Acharya R et al. *JAMA Network Open.*

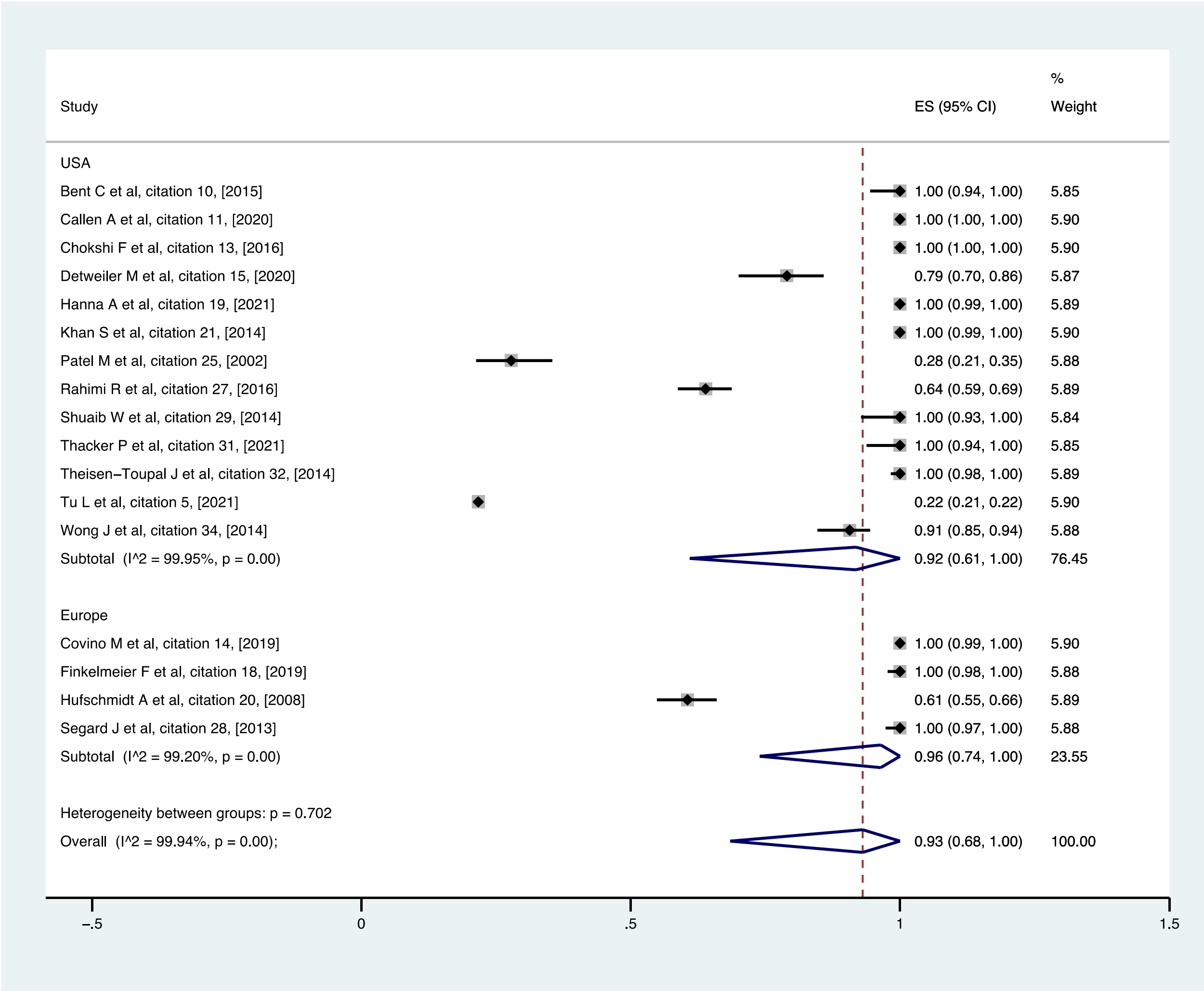


**eFigure 5.** The Proportion of Computerized Tomography of Head (CTH) in Patients With Altered Mental Status (AMS) Among Studies
ES= Effect size representing the proportion of CTH in AMS patients. The model used is the random effect model. USA= United States of America

© 2022 Acharya R et al. *JAMA Network Open.*
